# Nitrogen input by bamboos in neotropical forest: a new perspective

**DOI:** 10.7717/peerj.6024

**Published:** 2018-11-29

**Authors:** Maíra C.G. Padgurschi, Simone A. Vieira, Edson J.F. Stefani, Gabriela B. Nardoto, Carlos A. Joly

**Affiliations:** 1 Plant Biology Department, State University of Campinas, Campinas, São Paulo, Brazil; 2 Center for Environmental Studies and Research, State University of Campinas, Campinas, São Paulo, Brazil; 3 Department of Ecology, University of Brasilia, Brasilia, Brazil

**Keywords:** *Merostachys neesii*, Atlantic forest, Free-living biological nitrogen fixation, N cycling, Neotropical bamboo

## Abstract

**Background:**

Nitrogen (N) is an important macronutrient that controls the productivity of ecosystems and biological nitrogen fixation (BNF) is a major source of N in terrestrial systems, particularly tropical forests. Bamboo dominates theses forests, but our knowledge regarding the role of bamboo in ecosystem functioning remains in its infancy. We investigated the importance of a native bamboo species to the N cycle of a Neotropical forest.

**Methods:**

We selected 100 sample units (100 m^2^ each) in a pristine montane Atlantic Forest, in Brazil. We counted all the clumps and live culms of *Merostachys neesii* bamboo and calculated the specific and total leaf area, as well as litter production and respective N content. Potential N input was estimated based on available data on BNF rates for the same bamboo species, whose N input was then contextualized using information on N cycling components in the study area.

**Results:**

With 4,000 live culms ha^−1^, the native bamboo may contribute up to 11.7 kg N ha^−1^ during summer (January to March) and 19.6 kg N ha^−1^ in winter (July to September). When extrapolated for annual values, *M. neesii* could contribute more than 60 kg N ha^−1^y^−1^.

**Discussion:**

The bamboo species’ contribution to N input may be due to its abundance (habitat availability for microbial colonization) and the composition of the free-living N fixer community on its leaves (demonstrated in previous studies). Although some N is lost during decomposition, this input could mitigate the N deficit in the Atlantic Forest studied by at least 27%. Our findings suggest that *M. neesii* closely regulates N input and may better explain the high diversity and carbon stocks in the area. This is the first time that a study has investigated BNF using free-living N fixers on the phyllosphere of bamboo.

## Introduction

Woody bamboos are typical plants in many tropical forests (Humboldt & Bonpland, 1907; Judziewicz et al., 1999). Their rapid growth under intense levels of light (Cirtain, Franklin & Pezeshki, 2009) and leaves with relatively low carbon cost and high photosynthesis rates (Montti et al., 2014; Yang et al., 2014) result in the widespread occurrence of these plants in forests (Judziewicz et al., 1999). Bamboo density effects the dynamics and structure of forests (Tabarelli & Mantovani, 2000; Griscom & Ashton, 2003; Giordano, Sánchez & Austin, 2009; Rother, Rodrigues & Pizo, 2009; Lima et al., 2012), serving as a resource for different animals (Reid et al., 2004; Areta, Bodrati & Cockle, 2009; Hilário & Ferrari, 2010; Cestari & Bernardi, 2011). Although it is unclear whether they influence ecosystem function, studies in this regard have increased and demonstrate the role of bamboo in recovering soil fertility (Christanty, Kimmins & Mailly, 1997), especially nitrogen (Singh & Singh, 1999; Embaye et al., 2005; Fukuzawa et al., 2006; Watanabe & Fukuzawa, 2013; Shiau et al., 2017; Borisade & Odiwe, 2018).

Nitrogen (N) controls the productivity and composition of plant species (Townsend et al., 2011) and is a limiting factor in many tropical forests (Tanner, Vitousek & Cuevas, 1998), making N recycling via litter decomposition a key resource in these forests (Vitousek & Sanford, 1986; Kuruvilla, Jijeesh & Seethalakshmi, 2014; Borisade & Odiwe, 2018). The rapid growth of bamboo, its overabundance and biomass (Yang et al., 2014) contribute to nutrient pumping, that is, nutrients leached into the soil are deposited at the surface as bamboo litterfall (Christanty, Kimmins & Mailly, 1997). However, its intensity depends primarily on the lignin:N (Tripathi et al., 2006) and silicate:N ratios of leaves (Watanabe & Fukuzawa, 2013). In other words, the decomposition rate is greater when N content is high and lignin or silicate levels are low (Tripathi et al., 2006; Watanabe & Fukuzawa, 2013).

In an agroforestry system in Indonesia, the N content in bamboo litterfall varied from 28.2 to 45.2 kg ha^−1^ (Mailly, Christanty & Kimmins, 1997), with concentrations of 5 to 57 kg N ha^−1^ y^−1^ in other Asian ecosystems (Joshi, Sundriyal & Baluni, 1991; Mailly, Christanty & Kimmins, 1997) and 33.2 (Kuruvilla, Jijeesh & Seethalakshmi, 2014; Kuruvilla, Jijeesh & Seethalakshmi, 2016) to 79 kg N ha^−1^ in India (Singh & Singh, 1999). Nevertheless, these figures pale in comparison to the 115 kg N ha^−1^recorded for *Yushania alpina* in Ethiopia (Embaye et al., 2005). Although there are exceptions (Singh & Singh, 1999; Tripathi et al., 2006), bamboo litter typically exhibits a high N concentration (Joshi, Sundriyal & Baluni, 1991; Embaye et al., 2005; Watanabe & Fukuzawa, 2013; Kuruvilla, Jijeesh & Seethalakshmi, 2014; Kuruvilla, Jijeesh & Seethalakshmi, 2016; Borisade & Odiwe, 2018), but may also contain high lignin and silicate levels, meaning the N in its litter tends to be released gradually over an extended period (Tripathi et al., 2006; Watanabe & Fukuzawa, 2013; Borisade & Odiwe, 2018).

In addition to N recycling, biological nitrogen fixation (BNF) is an important pathway for N input in ecosystems (Hedin et al., 2009). Studies have shown that free-living BNF fixers in litter and those associated with the aerial parts of plants play a vital role in total N inputs in tropical forests (Bentley, 1987; Benner et al., 2007; Reed, Cleveland & Townsend, 2011). The leaf surfaces (phyllosphere) in these forests harbor a wide range of bacteria (Lambais et al., 2006; Fürnkranz et al., 2008; Lambais, Lucheta & Crowley, 2014), many of which are N fixers and dictate the patterns of N fixation rates (Reed, Cleveland & Townsend, 2011; Rigonato et al., 2016).

In the Brazilian Atlantic Forest (AF), Gómez (2012) found a high level of bacterial diversity in the phyllosphere of *Merostachys neesii* (Poaceae: Bambusoideae), including groups of putative free-living diazotrophs that account for a significant amount of N fixation. Studying the same bamboo species, Rigonato et al. (2016) reported a high abundance of cyanobacteria from the diazotrophic order Nostacales. In this AF area, unlike several other studies (Tabarelli & Mantovani, 2000; Griscom & Ashton, 2003; Lima et al., 2012), the presence of *M. neesii* in a pristine montane forest does not seem to alter the overall forest structure and diversity (Padgurschi et al., 2011), carbon and nitrogen stocks (Vieira et al., 2011) or tree biomass (283.2 Mg ha^−1^) (Alves et al., 2010). The presence of *M. neesii,* showing evidence of free-living diazotrophs on its leaves, suggests that these plants have efficient mechanisms to cope with potential nutrient limitations in acidic dystrophic soils (Martins et al., 2015).

However, disturbances resulting from land use changes may cause an unusual overabundance of native plants (Pivello et al., 2018), including bamboos, which may also respond positively to CO_2_ concentration and produce additional biomass (Grombone-Guaratini et al., 2013). Moreover, human activities, such as urbanization and industrialization, produce significant atmospheric N pollution (Souza et al., 2015). These N additions can have a substantial effect on decomposition rates since they can indirectly shift the microbial community (Agren, Bosatta & Magill, 2001). Thus, investigating the influence of bamboo on N cycling is key to understanding and predicting ecosystem responses to global changes.

The present paper sought to provide insights on the role of bamboo (*M. neesii*) in the functioning of a Neotropical forest. The major objectives were: (i) to assess the abundance of bamboo in an Atlantic Forest area; (ii) to understand the amount of N added to the system by *M. neesii* via free-living diazotrophs in its phyllosphere; (iii) to calculate the amount of N that returns to the system through *M. neesii* litter; and (iv) to contextualize the N added by *M. neesii* using information about N cycling components in the study area.

## Materials and Methods

### Study area

**Figure 1:**
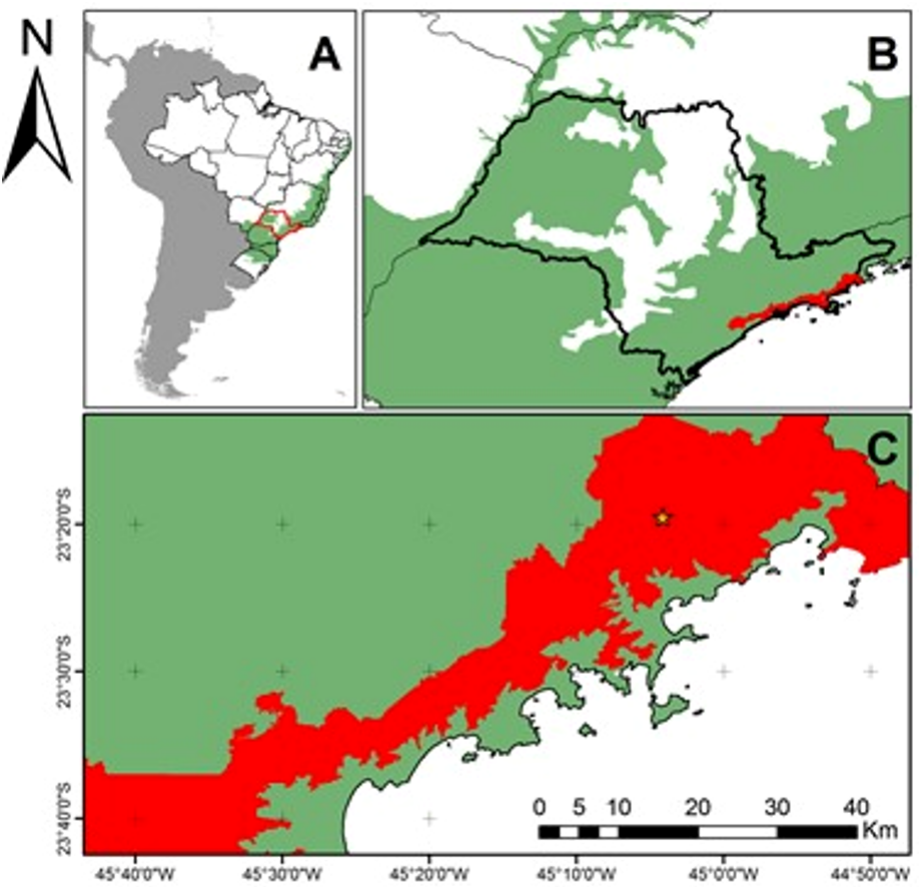
Location of the study area in the context of the Brazilian Atlantic Forest Domain and Serra do Mar State Park (45°W04′34″23°S17′24″). Brazilian Atlantic Forest Domain (green) and Serra do Mar State Park (red) (PESM in Portuguese). (A) South America with a focus on Brazil. In green: Atlantic Forest Domain; (B) São Paulo State, SE Brazil. In red: PESM; (C) Study area (yellow star) in the context of PESM.

The study was conducted in an Atlantic Forest region in northeastern São Paulo state, Brazil, in the Serra do Mar State Park (PESM in Portuguese) (Fig. 1). We selected 100 sample units (100 m^2^ each) within previously established permanent plots (Joly et al., 2012). The physiognomy is pristine montane Atlantic Forest (1,000 m a.s.l.), with a humid subtropical climate (Cfa and Cfb), average annual temperature of 21 °C, average annual rainfall of 2,180 mm, and no dry season (Salemi et al., 2013). A dense fog covers the region almost daily, especially in winter. The soil order is Inceptisol (United States Department of Agriculture taxonomy), with low pH (≈3.8) and fertility, and high aluminum saturation (Martins et al., 2015). Both aboveground biomass (283.2 Mg ha^−1^) (Alves et al., 2010) and floristic diversity (∼200 tree species ha^−1^) (Padgurschi et al., 2011) are high (Joly, Metzger & Tabarelli, 2014). The most abundant families are Arecaceae, Myrtaceae, Lauraceae and Sapotaceae (Padgurschi et al., 2011).

### Bamboo species: density, leaf area and litterfall

**Figure 2:**
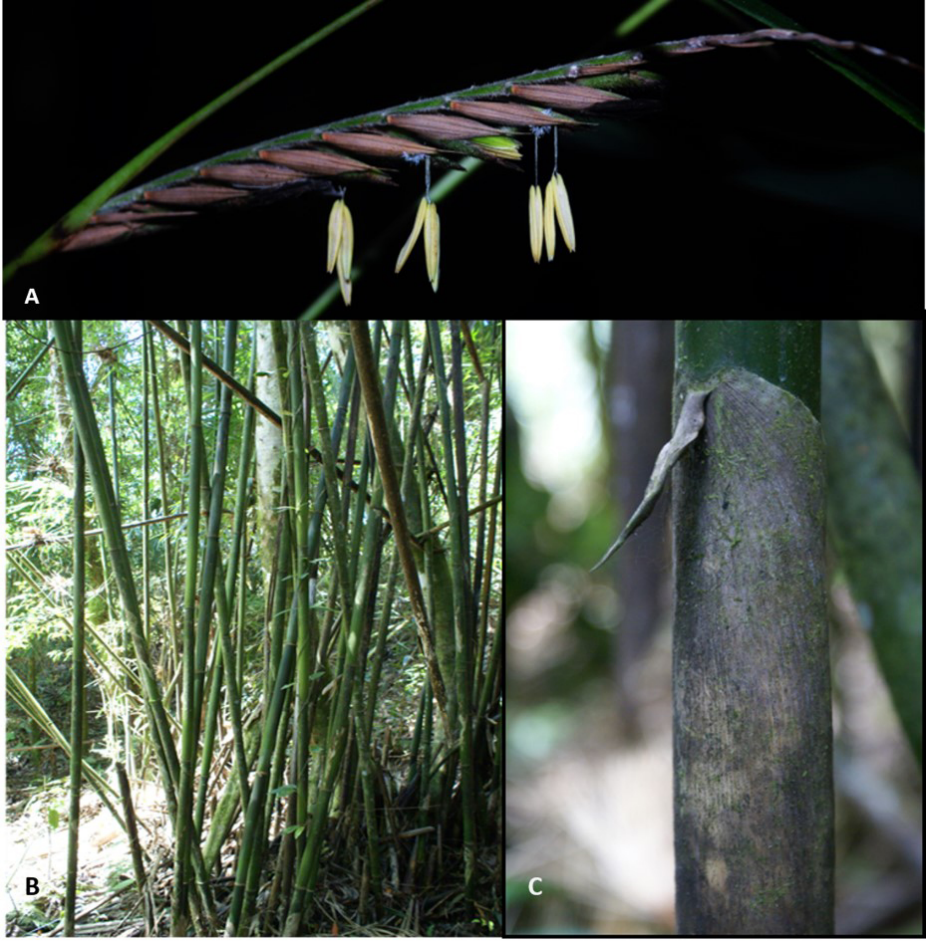
*Merostachys neesii* Rupr. (Poaceae: Bambusoideae), a native woody bamboo in a pristine montane forest (Atlantic Forest), Brazil. (A) Flowers at anthesis; (B) Detail of a clump in the study area; (C) Detail of the culm leaf of *M. neesii*, a characteristic of this species. Photos: MCG Padgurschi.

*Merostachys neesii* Rupr. (Poaceae: Bambusoideae), a native species of the Brazilian Atlantic Forest (Fig. 2), prefers humid, high-altitude environments (Judziewicz et al., 1999). All the clumps and live culms in the 100-sample units were counted (culm density) and culm density was compared against the highest density species in the area (*Euterpe edulis* Mart. Arecaceae—Padgurschi et al., 2011).

Habitat availability (bamboo leaf area) was estimated in order to determine N input by free-living diazotrophs in the phyllosphere. We calculated the total bamboo leaf area (*LA*_*t*_) based on (i) culm density; (ii) leaf biomass per culm (*L*_*b*_*)*; and (iii) specific leaf area (*SLA*). *L*_*b*_ was previously determined by MCG Padgurschi, TS Reis, LF Alves, SA Vieira, CA Joly (2018, unpublished data) via destructive harvesting of 20 healthy culms around the study area (*L*_*b*_ = 506 g; 95% bootstrap, confidence interval: 316.2 and 701.2 were the lower/upper limits, respectively). For *SLA,* we randomly chose 50 bamboo leaves in the field, dried at 65 °C until constant weight, weighed to obtain the dry weight, and the leaf area was calculated using an LI-3100 area meter (LI-COR, Lincoln, Nebraska, USA). The leaf dry weight and leaf area (*n* = 50) were then used to calculate SLA. Leaf area per culm (*LA*_*c*_) was determined as follows:

(1) \begin{eqnarray*}L{A}_{c}={L}_{b}\ast SLA\end{eqnarray*}

and total bamboo leaf area (*LA*_*t*_) (m^2^ ha^−1^) by:

(2) \begin{eqnarray*}L{A}_{t}= \frac{L{A}_{c}\text{*n culms}}{10000} \end{eqnarray*}

where “n culms” is the culm density within the sample units.

Among the 100 sample units, we randomly selected 40 to install circular litter traps (0.22 m^2^ each). The traps were made of malleable plastic pipes with nylon mesh (2 mm) and supported by PVC pipes about 1 m above the ground. The content of the traps was collected twice a month over a year, from April 2014 to April 2015, sufficient time to capture this variable (Malhi, Doughty & Galbraith, 2011). For each collection, the bamboo leaves were separated, dried (at 65 °C until constant weight) and weighed to obtain dry weight. We calculated the production of bamboo litterfall in accordance with Sylvestre & Rosa (2002):

(3) \begin{eqnarray*}\mathrm{LP}= \frac{ \left( \frac{\sum \mathrm{MA}\ast 10,000}{\mathrm{CA}} \right) }{1000} \end{eqnarray*}

where LP = annual litter production (kg ha^−1^y^−1^); MA = average monthly litter production (kg ha^−1^); CA = litter collector area (m^2^). For N chemical analysis of the bamboo leaves, we randomly selected three samples for each season (summer, fall, winter, spring) and ground them to obtain a compound sample per season (results are expressed in kg N ha^−1^). The analysis was performed at the Soil and Plant Laboratory (LAGRO), in São Paulo, Brazil, using the Kjeldahl method of N determination. The study was performed with permits COTEC/IF 010.323/2013, 002.766/2013 and 010.631/2013 and IBAMA/SISBIO #33217.

### Estimating N input by free-living N fixers in the *M. neesii* phyllosphere

To estimate N input by free-living diazotrophs on bamboo leaves, we used BNF rates previously recorded in the *M. neesii* phyllosphere at the same site studied here (Gómez, 2012). Gómez (2012) estimated BNF rates by acetylene reduction activity (ARA) based on a theoretical conversion ratio of 3:1 (reduction of three acetylene moles for each N mole fixed) (Hardy et al., 1968). The BNF rate in the bamboo phyllosphere was 64.25 ng N cm^−2^ h^−1^in winter and 34.78 ng N cm^−2^ h^−1^ in summer and, given the significant difference between these two values (Gómez, 2012), calculations for each season were performed separately.

Since light and temperature are important variables that affect microbial activity (Bentley, 1987; Reed, Cleveland & Townsend, 2011), we also considered the differences in hours of light during seasons. As such, based on available photosynthetically active radiation (PAR) data provided by the Climate and Biosphere Laboratory/Dept. of Atmospheric Sciences/University of São Paulo, bootstrapping (4,000 resamplings) was carried out to obtain the median and lower/upper limits of PAR (Table 1). We used the number of hours around the PAR median added to the lower/upper limits (828 ± 70 µmol m^−2^ s^−1^ in summer; 711.24 ± 55 µmol m^−2^ s^−1^ in winter; 95% confidence intervals) (Table 1). Finally, N fixing potential was estimated (*N*_*f*_ expressed in kg N ha^−1^y^−1^) as follows:

(4) \begin{eqnarray*}{N}_{f}= \frac{ \left( BNF\ast L{A}_{t} \right) \ast {H}_{l}}{1{0}^{12}} \end{eqnarray*}

where *H*_*l*_ is the hours of light in summer or winter (Table 1).

**Table 1:** Meteorological data for the study area in 2010, the same year as the BNF rates data. Hours of light/day, hours with photosynthetically active radiation (PAR) around the PAR median of the respective seasons months; PAR min. and PAR max., photosynthetically active radiation minimum and maximum, respectively, recorded for that season; Median calculated from bootstrapping (4,000 resampling) with the 95% confidence interval in parentheses.

Season	Light (hours/day)	PAR min. (µmol m^−2^ s^−1^)	PAR max. (µmol m^−2^ s^−1^)	**Median** (µmol m^−2^ s^−1^)	Mean temperature (°C)	Accumulated rainfall (mm)
Summer	9	4.47	2,670.3	828.0 (±70)	19.3	380.4
Fall	8	5.76	2,261.7	773.9 (±41)	13	417.4
Winter	8	3.78	2,064	711.2 (±55)	12.6	295.5
Spring	9	13.92	2640	602.6 (±40)	12.9	692

### N cycling

To contextualize the estimated N input mediated by *M. neesii*, data on the N cycling in the Atlantic Forest were obtained from the literature. The two dominant N input pathways (Hedin et al., 2009) considered were symbiotic BNF (Manarin, 2012) and total atmospheric N deposition (Groppo, 2010), in addition to the free-living N fixers on bamboo leaves (this study).

In terms of N required by the system (demand), we used litterfall to predict net primary productivity (NPP). The NPP fraction allocated to leaves influences litterfall rates, making it a good predictor of productivity in neotropical forests when the main components of NPP cannot be measured (Malhi, Doughty & Galbraith, 2011). Based on this principle, we used the literature data on ecosystem litter production (5.5 Mg ha^−1^ y^−1^—Sousa Neto et al., 2011) and the N content of the litter (1.72%—Vieira et al., 2011), as well as bamboo litter with its respective nitrogen concentration (see the “Bamboo species: density, leaf area and litterfall” section for details). The N content of litter is equivalent to the minimum amount required for tree and bamboo growth, since plants reallocate nutrients before leaf abscission, meaning litter exhibits lower N levels when compared to live leaves (Chapin III et al., 1987; Tripathi et al., 2006). The annual production of fine roots (<2 mm) was considered representative of demand. These roots represent at least twice as much carbon and nitrogen stock as that found aboveground in the AF (Vieira et al., 2011). Fine root production of 10 Mg ha^−1^ y^−1^ (Silva, 2015) and N content of 1.3% (Sousa Neto et al., 2011) were used.

Finally, riverine transport and N_2_O and NO losses via soil emissions were included as outputs (Groppo, 2010; Sousa Neto et al., 2011; Ghehi et al., 2013). The NO emission we presented here is based on models developed for a tropical highland forest (Ghehi et al., 2013) similar to the study area, as follows: (i) pristine montane forest (1,000 m a.s.l.); (ii) 2,000 mm y^−1^ of rainfall; (iii) presence of bamboo; (iv) pH 3.8 (Ghehi et al., 2013; Martins et al., 2015). All analyses and graph were performed using R environment (R Core Team, 2014).

## Results

A total of 579 clumps ha^−1^ and 4,000 live culms ha^−1^ of *M. neesii* bamboo were counted. The specific leaf area (SLA) was 204.4 cm^2^ g^−1^ (95% bootstrap confidence interval: 196.7/210.2 lower/upper limits, respectively) which, by applying equation one, resulted in *LA*_*c*_ = 10.3 m^2^ and 4.1 ×10^4^ m^2^ ha^−1^ of total leaf area (*LA*_*t*_*)* for microbial colonization. These and other data are shown in Table 2.

**Table 2:** Traits of *M. neesii* and its contribution to nitrogen input in a pristine montane Atlantic Forest, São Paulo State, Brazil. Values in parenthesis are lower/upper limits of 95% confidence intervals obtained by bootstrapping (1,000 resampling).

***Merostachys neesii*Traits**	
Density (clumps ha^−1^)	579
Culms (ha^−1^)	4,000
(*L*_*w*_*)* (g)	0.11 (0.10–0.12)
*LA* (cm^2^)	23.2 (21.5–25.2)
*SLA* (cm^2^ g^−1^)	204.4 (196.7–210.2)
*LA*_*c*_ (m^2^)	10.3
*LA*_*t*_ (m^2^ ha^−1^)	4.1 × 10^4^
N fixed (kg N ha^−1^)—summer	11.7
N fixed (kg N ha^−1^)—winter	19.6
N content in bamboo litterfall (%)	1.65

**Notes.**

*L*_*w*_ Leaf dry weight *LA* Leaf area *SLA* Specific leaf area *LA*_c_ Leaf area per culm (estimated from Eq. (1) *LA*_t_ Total bamboo leaf area (estimated from Eq. (2) N fixed Total nitrogen fixed on bamboo phyllosphere during summer (Jan., Feb., Mar) and winter (Jul., Aug., Sep.) N content in bamboo litterfall % of nitrogen in bamboo leaves from litter

*M. neesii* can contribute up to 11.7 kg N ha^−1^ in summer (January to March), and 19.6 kg N ha^−1^ in winter (July to September), via free-living diazotrophs on its phyllosphere. When these values were extrapolated on an annual basis, *M. neesii* contributed more than 60 kg N ha^−1^y^−1^, representing a decline of at least 27.8% in the N deficit of the AF we studied (Table 3).

**Table 3:** Estimates of N inputs, demand and outputs in the Atlantic Forest studied. Except for NO soil emission, all the data were obtained from the Atlantic Forest area studied.

	**Reference**	**Biome**	**Compartment**	**Nitrogen(kg N ha**^−1^**y**^−1^**)**
Inputs	Groppo (2010)	Atlantic Forest, Brazil	N_total_(N-N_inorg_+N-N_org_) ^a^	2.8
	Manarin (2012)	Atlantic Forest, Brazil	BNF by legume trees	0.2
	This study	Atlantic Forest, Brazil	free-living BNF (bamboo leaves)	62.6
			**Total**	**65.6**
Demand	Sousa Neto et al. (2011 ), Vieira et al., 2011	Atlantic Forest, Brazil	Tree growth	86.1
	This study	Atlantic Forest, Brazil	Bamboo growth	8.9
	Sousa Neto et al. (2011)Silva (2015)	Atlantic Forest, Brazil	Fine root (<2 mm)	130.0
			**Total**	**225.0**
Outputs	Groppo (2010)	Atlantic Forest, Brazil	Riverine transport	0.6
	Sousa Neto et al. (2011)	Atlantic Forest, Brazil	N_2_O soil emission	0.8
	Ghehi et al. (2013)	Tropical Highland Forest, Rwanda	NO soil emission	2.0
			**Total**	**3.4**
Total				**−162.7**

**Notes.**

a Value referring to the wet deposition of N in the study area. The value presented refers to the average for 2008 and 2009.

**Figure 3:**
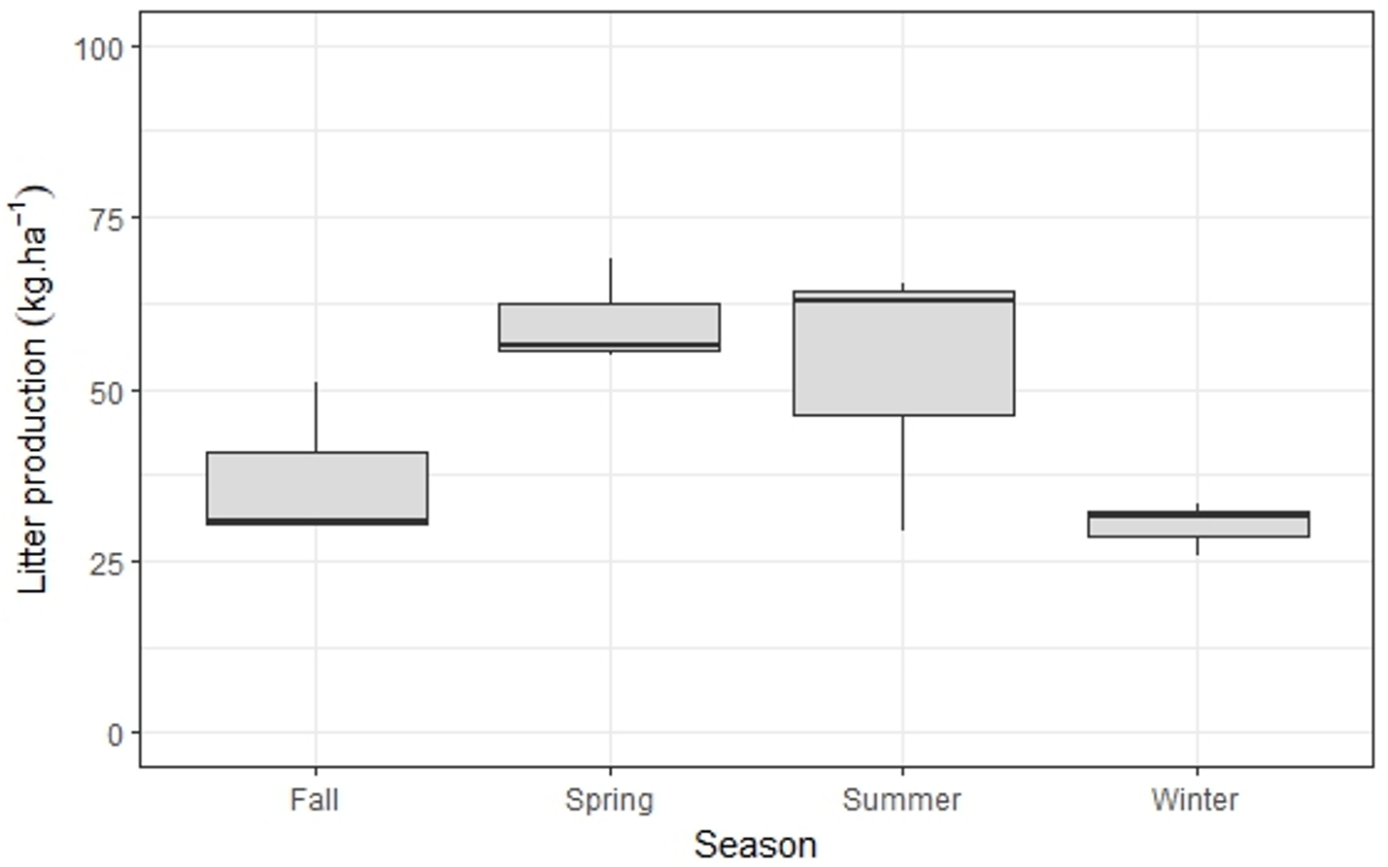
Seasonal variation of *M.neesii*’s litter production in the pristine montane Atlantic Forest, Brazil. Significantly higher values are found during summer/spring when compared to fall/winter (*p* < 0.001).

Annual bamboo litter production was 540 kg ha^−1^y^−1^, with significantly higher values in summer/spring when compared to fall/winter (*p* < 0.001) (Fig. 3). The N content in this litter fraction was 1.65% (Table 2); as such, the minimum N requirement for bamboo growth is 8.9 kg ha^−1^y^−1^ (Table 3).

## Discussion

Bamboo is important in the recovery of soil physiochemical properties (Christanty, Kimmins & Mailly, 1997; Embaye et al., 2005; Shiau et al., 2017), soil redevelopment (Singh & Singh, 1999) and soil nutrients, especially N (Fukuzawa et al., 2006; Watanabe & Fukuzawa, 2013; Shiau et al., 2017; Borisade & Odiwe, 2018). Its rapid growth and abundance (Yang et al., 2014) may contribute to nutrient pumping, whereby nutrients leached deep into the soil are deposited at the surface as bamboo litterfall (Christanty, Kimmins & Mailly, 1997).

Although the bamboo density observed here (Table 2) is lower than that found in India (Joshi, Sundriyal & Baluni, 1991; Tripathi & Singh, 1994; Christanty, Kimmins & Mailly, 1997; Singh & Singh, 1999), China (Wang et al., 2006) and Ethiopia (Embaye et al., 2005), it is similar to that reported in other bamboo forests in the Neotropics (Londoño & Peterson, 1991; Guilherme et al., 2004; Griscom & Ashton, 2006; Rockwell et al., 2014). The abundance and biomass of *M. neesii* (MCG Padgurschi, TS Reis, LF Alves, SA Vieira, CA Joly, 2018, unpublished data) provide a substantial habitat (leaf area) for microbial colonization (Table 2) which, when combined with the composition of the free-living bacterial community on its phyllosphere, may influence BNF rates (Benner et al., 2007; Reed, Cleveland & Townsend, 2011).

*M. neesii* exhibits higher cyanobacteria abundance and a larger number of diazotrophs affiliated to the order Nostocales (Rigonato et al., 2016) than *E. edulis* and other species from the same area (Gómez, 2012). Its phyllosphere harbored high annual BNF rates (∼60 kg N ha^−1^ y^−1^), almost equal to the rate reported for evergreen tropical forests (Reed, Cleveland & Townsend, 2011), but significantly higher than those observed for *Spathacanthus hoffmannii* (Acanthaceae), *Chamaedorea tepejilote* (Arecaceae), *Brosimum utile* (Moraceae), *Caryocar costaricense* (Caryocaraceae), *Staminodella manilkara* (Sapotaceae), *Qualea paraensis* (Vochysiaceae) and *Schizolobium parahybum* (Fabaceae) (between 0.035 and 5 kg N ha^−1^y^−1^—Freiberg, 1998; Reed, Cleveland & Townsend, 2008).

N input by bamboo could mitigate the N deficit in the AF we studied by at least 27% (Table 3), where, in addition to the low occurrence of tree legumes (Padgurschi et al., 2011), the symbiotic BNF rate (0.2 kg N ha^−1^ y^−1^—Manarin, 2012) is lower than that reported for the Amazon forest (Nardoto et al., 2014) and Costa Rica (Sullivan et al., 2014). Symbiotic BNF in mature tropical forests may not be as important as previously believed (Sullivan et al., 2014; Nardoto et al., 2014), making bamboo input particularly relevant, since the N demand of trees, bamboos and fine roots is at least 225 kg N ha^−1^ y^−1^ (Table 3). This is a minimum requirement, since only trees with diameter at breast high (DBH) ≥ 5 cm are included, with other life forms (such as epiphytes and lianas) excluded from the inventory data (Joly et al., 2012).

Despite the N input of bamboo, N demand is high in the system studied here (Table 3) and as a result, litterfall decomposition plays an important role in nutrition budgeting (Vitousek & Sanford, 1986; Kuruvilla, Jijeesh & Seethalakshmi, 2014; Borisade & Odiwe, 2018). The annual litter production of *M. neesii* (540 kg ha^−1^y^−1^) is lower than that of several tropical and subtropical bamboo species, except for *Dendrocalamus strictus* (580 kg ha^−1^—Joshi, Sundriyal & Baluni, 1991) and *Sasa senanensis* (600 kg ha^−1^y^−1^—Watanabe & Fukuzawa, 2013).

In an agroforestry system in Indonesia, the litterfall of different species of the genus *Gigantochloa* ranged from 3 to 4.7 Mg ha^−1^ (Mailly, Christanty & Kimmins, 1997); in an Ethiopian forest, the litterfall of *Y. alpina* was 8 Mg ha^−1^y^−1^ (Embaye et al., 2005); 1.2 and 1.9 Mg ha^−1^ were recorded in Japan for *Sasa kurilensis* (Tripathi et al., 2006), and 2.9 and 6.9 Mg ha^−1^ in India (Kuruvilla, Jijeesh & Seethalakshmi, 2014; Kuruvilla, Jijeesh & Seethalakshmi, 2016) (Singh & Singh, 1999). However, since the N content of *M. neesii* litter (1.6%) was similar to that reported in other studies (1.2% by Joshi, Sundriyal & Baluni, 1991, 1.4% by Embaye et al., 2005, 1.4% by Watanabe & Fukuzawa, 2013, 1.5% by Kuruvilla, Jijeesh & Seethalakshmi, 2014, 1.7% by Kuruvilla, Jijeesh & Seethalakshmi, 2016, 1.7% by Borisade & Odiwe, 2018, 0.7% by Singh & Singh, 1999, 0.9% by Mailly, Christanty & Kimmins, 1997 and 1% by Tripathi et al., 2006), the final amount of N generated from bamboo litter in each system depends on the annual amount of litter (a total of 8.9 kg N ha^−1^y^−1^ was reported in this study) .

Finally, it is well known that high N levels and low lignin or silicate concentrations in leaves increase the decomposition rate of leaf litter (Tripathi et al., 2006; Watanabe & Fukuzawa, 2013). The leaf lignin content in different bamboo species ranges from 25% (Borisade & Odiwe, 2018) to more than 40% (Tripathi et al., 2006; Borisade & Odiwe, 2018), with the same observed for silicate (around 20%) (Watanabe & Fukuzawa, 2013). As such, it is expected that the N in bamboo litter in the AF is released gradually (Tripathi et al., 2006; Borisade & Odiwe, 2018) over a period of 3 years or more (Watanabe & Fukuzawa, 2013).

## Conclusion

Our findings suggest that the N fixed by free-living BNF associated with *M. neesii* plays a key role in the functioning of the neotropical forest. This may explain the high diversity (Padgurschi et al., 2011), carbon and nitrogen stocks (Vieira et al., 2011) and biomass (283.2 Mg ha^−1^) (Alves et al., 2010) found in the same AF area (Joly, Metzger & Tabarelli, 2014), contradicting previous studies (Lima et al., 2012; Grombone-Guaratini et al., 2014). Nonetheless, disturbances resulting from human activities such as industrialization and land use changes may increase bamboo abundance (Pivello et al., 2018; Grombone-Guaratini et al., 2013). Thus, the role of bamboo in the overall N cycle in neotropical forests is vital to understanding ecosystem responses to global change.

##  Supplemental Information

10.7717/peerj.6024/supp-1 Supplemental Information 1 Raw data: bamboo traits data used to calculate the nitrogen fixation on its phyllosphere

## Additional Information and Declarations

### Field Study Permissions

The following information was supplied relating to field study approvals (i.e., approving body and any reference numbers): The research was performed with permits COTEC/IF 010.323/2013, 002.766/2013 and 010.631/2013 and IBAMA/SISBIO #33217.

### Data Availability

The following information was supplied regarding data availability: The raw data are provided in a Supplemental File.
